# The Immune Response of Hemocytes of the Insect *Oncopeltus fasciatus* against the Flagellate *Phytomonas serpens*

**DOI:** 10.1371/journal.pone.0072076

**Published:** 2013-08-28

**Authors:** Thiago L. Alves e Silva, Luiz R. C. Vasconcellos, Angela H. Lopes, Thaïs Souto-Padrón

**Affiliations:** Instituto de Microbiologia Paulo de Góes, Centro de Ciências da Saúde, Bloco I, Universidade Federal do Rio de Janeiro, Ilha do Fundão, Rio de Janeiro, Brazil; Technion-Israel Institute of Technology Haifa 32000 Israel., Israel

## Abstract

The genus *Phytomonas* includes parasites that are etiological agents of important plant diseases, especially in Central and South America. These parasites are transmitted to plants via the bite of an infected phytophagous hemipteran. Despite the economic impact of these parasites, many basic questions regarding the genus *Phytomonas* remain unanswered, such as the mechanism by which the parasites cope with the immune response of the insect vector. In this report, using a model of systemic infection, we describe the function of *Oncopeltus fasciatus* hemocytes in the immune response towards the tomato parasite *Phytomonas serpens*. Hemocytes respond to infection by trapping parasites in nodular structures and phagocytizing the parasites. In electron microscopy of hemocytes, parasites were located inside vacuoles, which appear fused with lysosomes. The parasites reached the *O. fasciatus* salivary glands at least six hours post-infection. After 72 hours post-infection, many parasites were attached to the salivary gland outer surface. Thus, the cellular responses did not kill all the parasites.

## Introduction

Species of the genus *Phytomonas* are flagellates of the order Kinetoplastea, Trypanosomatidae family and are important etiological agents of lethal plant diseases, affecting crops of economic importance, such as coffee, cassava and coconut and palm oil, in Central and South America [1]. Depending on the species, *Phytomonas* spp. may be isolated from diverse vegetal parts such as latex, phloem, fruit sap and seed albumen of many plant families [1]. The transmission to plants occurs through the saliva of phytophagous hemipteran [2]. When these insects become infected while feeding on an infected plant, the parasites colonize the midgut and once a midgut infection is established, they cross the midgut epithelium, thereby gaining access to the hemocoel when the systemic infection occurs [1]–[3]. In the final step of insect colonization, parasites from the hemolymph bind to and invade the insect salivary glands [4], [5]. To successfully colonize and complete all developmental steps within the vector, the parasites must survive the insect immune response.

The insect immune response may be categorized in two types of interchangeable responses: the humoral and cellular responses [6]. The former includes the production of both antimicrobial peptides as well as reactive oxygen and nitrogen species. The humoral response also includes the activation of complex enzymatic cascades that regulate hemolymph coagulation, melanization and pathogen recognition [7]–[9]. The cellular responses in turn are mediated by hemocytes and include phagocytosis, nodulation and encapsulation [10]. Some events mediated by the humoral response depend on hemocyte activity.

In insects, the function of hemocytes in innate immunity has been extensively studied. Nodulation refers to the binding of multiple hemocytes to aggregate pathogens [11]. This response has been described to act against a wide range of pathogens, such as bacteria, fungi and protists [10]. In the hematophagous insect *Rhodnius prolixus*, hemocytes form nodules and aggregate the parasite *Trypanosoma rangeli* when the parasite colonizes the insect hemocoel [12]. In contrast, encapsulation refers to the binding of hemocytes forming layers on targets that are too large to be phagocytized, such as eggs of parasitoid wasps and nematodes [13], [14].

Phagocytosis is a widespread phenomenon among the animal kingdom [15]. In multicellular organisms, one of the most remarkable functions of phagocytosis is the protection against microorganisms. It is a complex process involving the recognition, engulfment and intracellular destruction of invading pathogens [15]. The phagocytic response occurs when a target binds to its cognate receptor on the cell surface, thereby inducing the activation of signal transduction pathways that culminate in the formation of a phagosome, which acts in an actin polymerization-dependent fashion [16]–[18]. Phagocytosis acts against a range of microorganisms in diverse experimental models [10] and also against parasites of medical importance such as *Plasmodium* spp, when these parasites invade the hemocoel of *Anopheles* mosquitoes [19]. Also *T. rangeli* is phagocytized by hemocytes of *R. prolixus* [12], [20].

Recently, the use of insects as experimental models to study host-parasite interactions has been validated as an excellent option to identify virulence factors associated with agents of important infectious diseases [21], [22]. In this work, we have investigated the cellular immune response of the hemipteran *Oncopeltus fasciatus* to *Phytomonas serpens* to shed light on an understudied aspect of the development of *Phytomonas* in their vectors. *O. fasciatus* is the natural vector of *Phytomonas elmassiani* and other lower trypanosomatids [5], which has been widely used as an experimental model to study the interaction with several species of trypanosomes [23], [24]. In this report, during the infection of *O. fasciatus* with *P. serpens*, these parasites multiply in the hemolymph and modify their morphology inducing long slender parasites, a common morphotype observed during the life cycle of *Phytomonas* species. Additionally, the infection induces the activation of cellular responses. These responses included an increase in the number of circulating hemocytes (hemocytes present in the hemolymph); the formation of nodules, which trap the parasites and adhere to insect organs removing parasites from the circulation and phagocytosis. The phagocytized parasites were within a parasitophorous-like vacuole, which fuses with previously gold-labeled lysosomes. Despite the induction of a cellular response, the parasites reach the salivary glands six hours post-infection (hpi) and increase in number at this site during the time course of the infection.

## Results

### Survival of *O. fasciatus* systemically challenged with *P. serpens*

**Figure 1:**
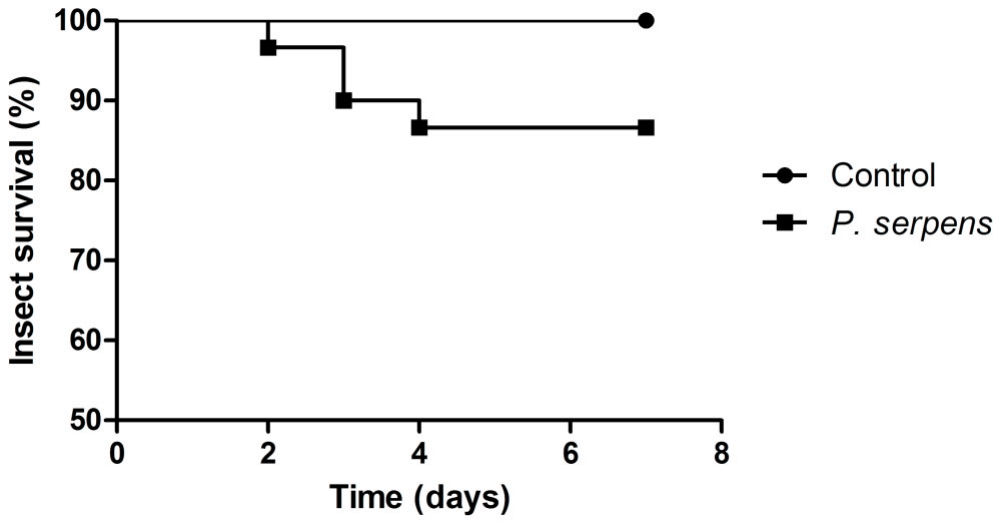
Survival of *P.* *serpens*-infected *O. fasciatus*. Groups of 25 adult insects were either infected with 5×10^4^ parasites or mock-infected with PBS and their survival was monitored twice a day. The mock-infected insects did not die due to the challenge. On the other hand, approximately 13% of the insects infected with parasites died, which was significant (P<0.05). The experiment was conducted twice.

To conduct the experimental infection, 5×10^4^ *P. serpens* was injected into the hemocoel of *O. fasciatus*. Within seven days after injection of *O. fasciatus*, 13% of the insects died (Fig. 1). Injection of PBS did not affect the viability of the insects. This sub-lethal dose was used in all experiments conducted here to understand the function of hemocytes during infection.

### *P. serpens* multiplies in *O. fasciatus* hemolymph and induces the long slender forms

**Figure 2:**
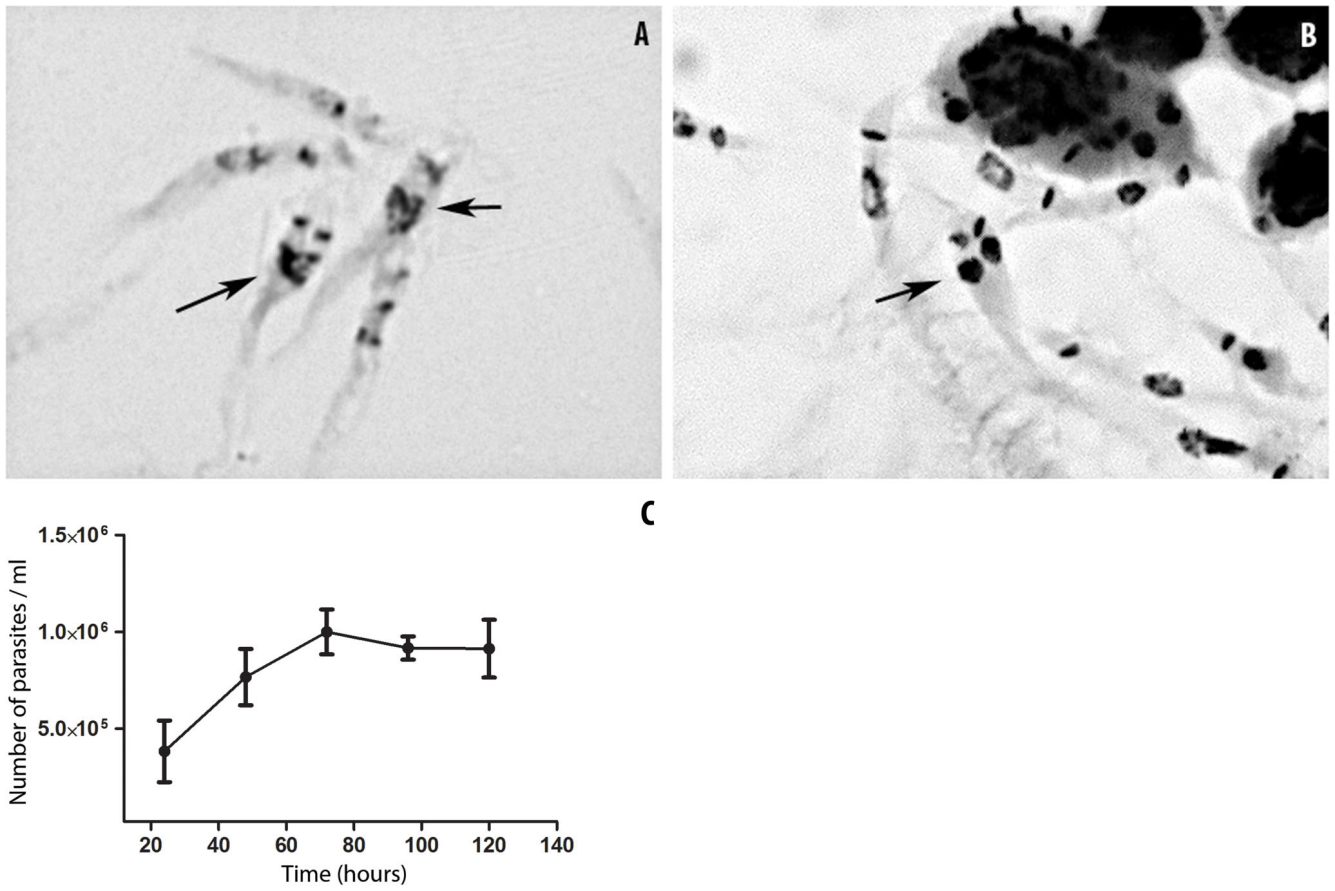
*P.* *serpens* multiplies in *O. fasciatus* hemolymph. (A and B) Promastigotes in the process of cell division were observed via Giemsa staining. A. 6 hpi; B. 24 hpi. The arrows indicate parasites undergoing division. (C) The number of flagellates in the hemolymph of *O. fasciatus* after the inoculation of 5×10^4^ parasites. Each point represents the mean ± standard error of five samples. Each sample was obtained by pooling together the hemolymph of at least three insects.

**Figure 3:**
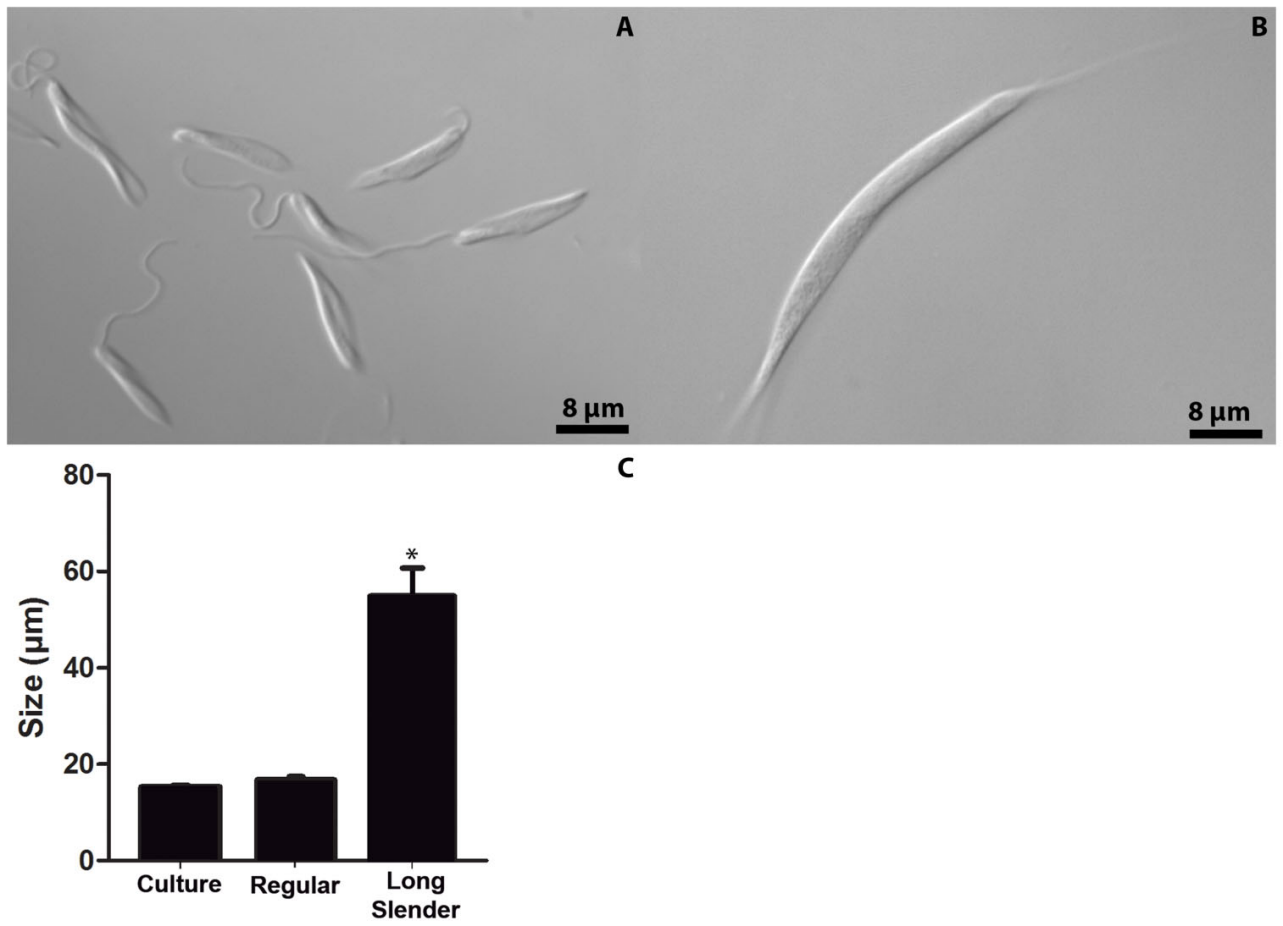
*P.* *serpens* displays various morphotypes within the hemocoel. (A and B) DIC of living parasites taken from: A. cell culture in stationary phase and B. hemolymph 72 hpi. (C) The mean length (mean ± standard error) of the parasite cell bodies isolated from culture and hemolymph 72 hpi. The asterisk indicates that the sample is significantly different from the other two groups (P<0.01). The statistics were performed using One-way ANOVA with Dunnett's post test.

The number of parasites in the hemolymph was determined at 24, 48, 72, 96 and 120 hpi. The parasites multiplied in the hemolymph (Fig. 2A and 2B). The parasites reached the peak of parasitemia at 72 hpi. At this time point, two populations of parasites were observed (Fig. 3A), a population with an average cell body length of 16.7 (±0.6) µm and long slender parasites with an average cell body length of 54.8 (±6.4) µm (Fig. 3B). As there is no definition for long slender forms in the literature, we considered as a long slender parasite any parasite with a cell body length over 30 µm. After 72 hours, approximately 40% of the parasites in the circulation and almost 100% of the parasites attached to the salivary glands were long slender promastigotes (Fig. 3B and 9C).

### The number of circulating hemocytes increases after challenging *O. fasciatus* with *P. serpens*

**Figure 4:**
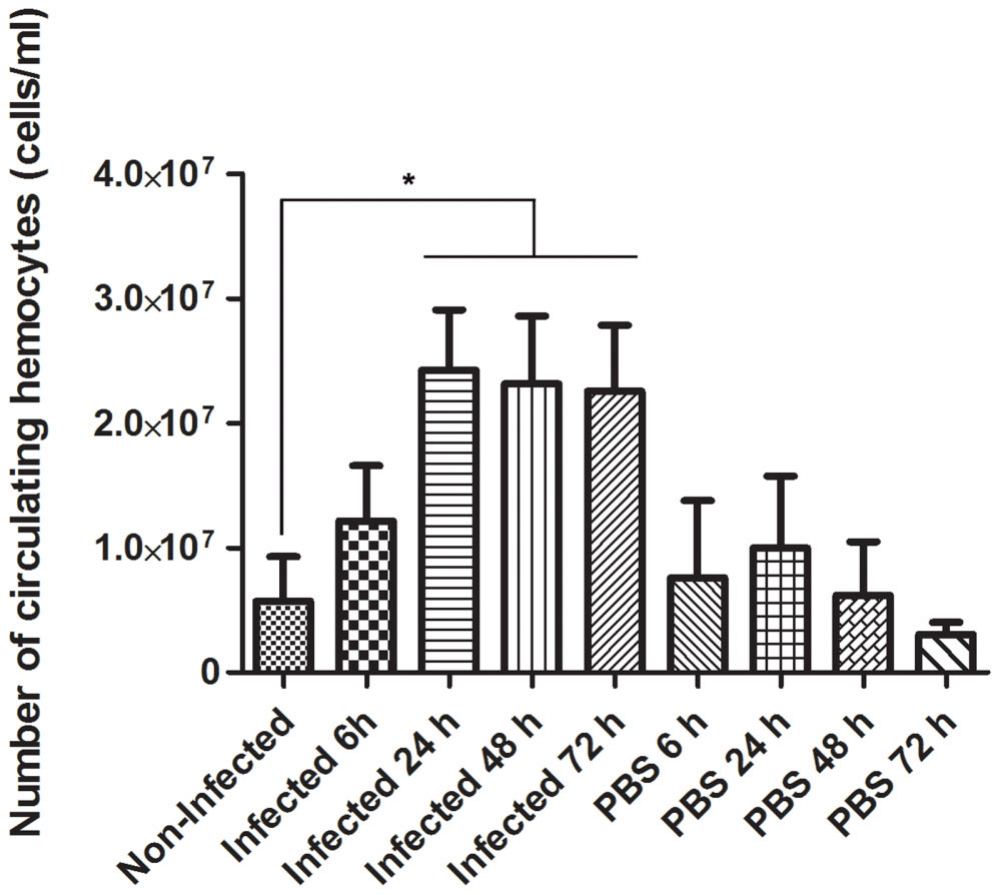
The number of circulating hemocytes in *O.* *fasciatus* hemolymph increases after infection. The insects (n = 24) were challenge with 5×10^4^ parasites and the cellular density of the hemocytes was determined using a Neubauer hemocytometer chamber. Each bar represents the mean ± standard error of eight samples and each sample was obtained by pooling together the hemolymph of three insects. Alternatively, the insects were mock challenged with PBS. The asterisk indicates that each one of the three bars (infected 24 h, infected 48 h and infected 72 h) has value significantly different from the value obtained for the hemocytes collected from hemolymph of non-infected insects, using One-way ANOVA with Dunnett's post test (p<0.05).

To investigate whether challenge with *P. serpens* induces a cellular immune response in *O. fasciatus*, the density of circulating hemocytes were determined (Fig. 4). When the insects were mock challenged with PBS, the hemocytes count did not change significantly compared with the control. Thus, the challenge of *O. fasciatus* with *P. serpens* induces an increase in hemocyte count.

### Hemocytes form nodules that trap *P. serpens*

**Figure 5:**
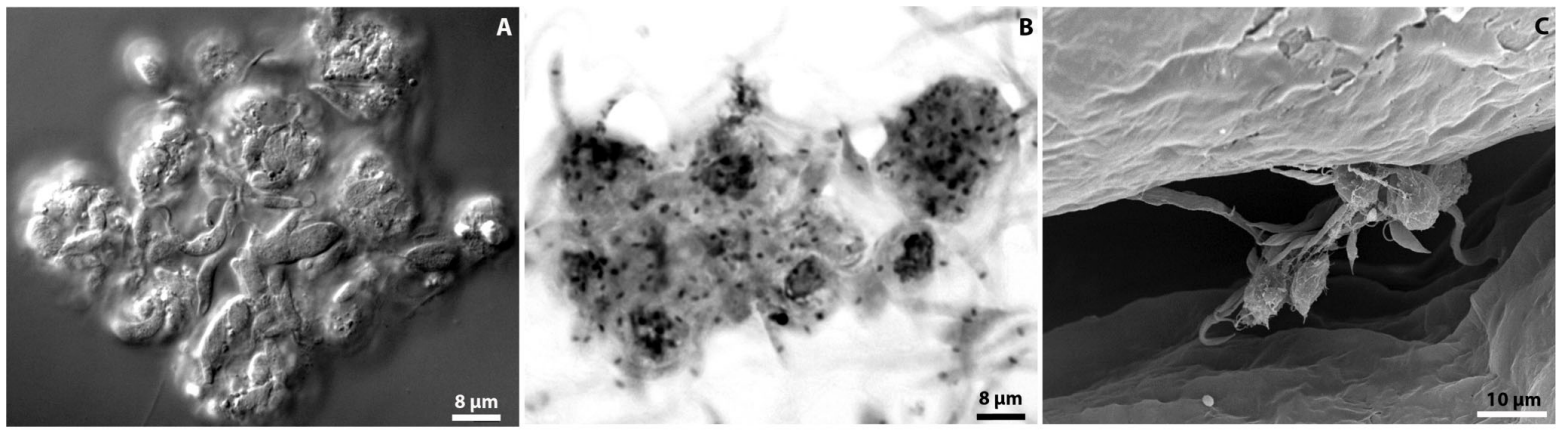
The challenge of *O.* *fasciatus* with *P. serpens* induces the formation of nodules. (A) Light microscopy of living hemocytes and parasites 6 h post-infection and (B) Giemsa-stained nodules 72 hpi. The scale bars are the same for both figures A and B, they represent 100× magnification; (C) Scanning electron microscopy of nodules 72 hpi. This figure is equivalent to 1,500× magnification.

To determine the mechanism by which hemocytes respond to *P. serpens* challenge, aliquots of hemolymph derived from infected insects were examined using light and scanning electron microscopy. Living parasites were trapped in nodular structures formed by hemocytes as early as 24 hpi (Fig. 5A and 5B). At the same post-infection period, nodules were also observed adhering to the surface of salivary glands (Fig. 5C).

### *P. serpens* is phagocytized by hemocytes and the number of hemocytes containing phagocytized parasites increases during the course of infection

**Figure 6:**
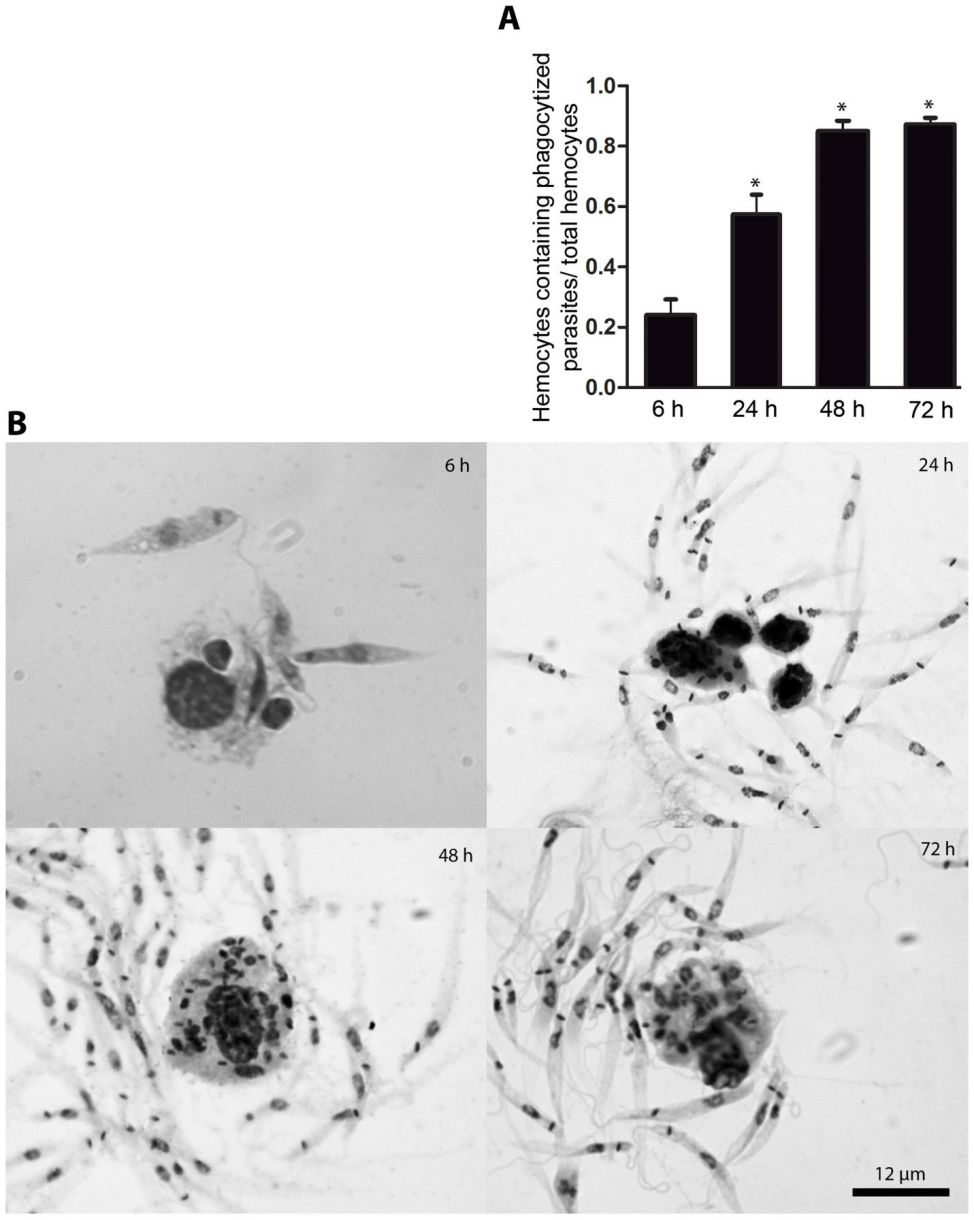
Hemocytes phagocytize *P.* *serpens*. (A) The ratio of hemocytes containing phagocytized parasites increased throughout the time course of the infection. Each point represents the mean ± standard error of eight samples; each sample was obtained by pooling together the hemolymph of three insects. The mean values were compared using One-way ANOVA with Dunnett's post test (p<0.05). The mean values of 24, 48 and 72 hpi differed significantly from the mean value of 6 hpi, as indicated by asterisks. (B) The number of parasites within the hemocytes increased during the infection. The hemocytes were fixed followed by Giemsa staining at 6, 24, 48 and 72 hpi.

At 6 hpi, 24% of total hemocytes contained phagocytized parasites. This number increased to 57.4% after 24 hpi, 85% after 48 hpi and 87% after 72 hpi (Fig. 6A). In Giemsa-stained preparations, parasites were observed within hemocytes at 6 hpi (Fig. 6B). The number of parasites within hemocytes increased with the increase in infection time. After 72 h, some hemocytes contained plenty of parasites and appeared damaged due the increased number of intracellular parasites (Fig. 6B). Therefore, the hemocytes actively take part in the host immune response during *P. serpens* infection and the parasite may multiply inside hemocytes.

### Ultrastructure of hemocytes containing phagocytized parasites

**Figure 7:**
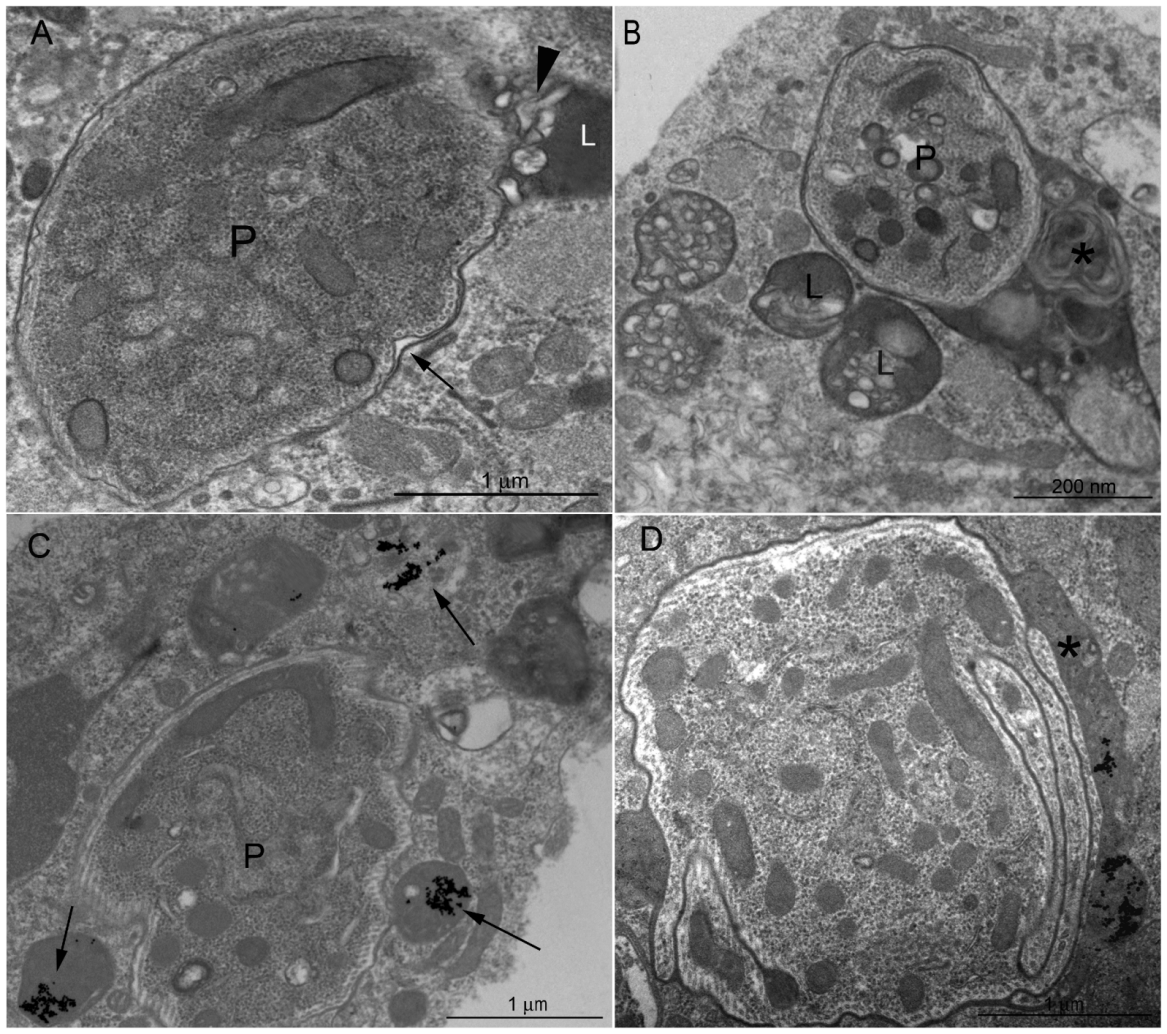
Intracellular fate of *P.* *serpens*. Infected hemocytes were analyzed at 6 hpi. (A) Parasites were observed in a tight PV, for which the membrane is indicated by the black arrows; (B) parasites were also observed in larger PV (asterisk) filled with the same structures (myelin figures, electron-dense matrix and lipid inclusions) regularly found in the lysosomes (L) of hemocytes; (C) lysosomes previously labeled with the BSA-gold complex (10 nm) were observed around the PV (black arrows). (D) In some sections, it was possible to observe gold particles inside the PV and close to the parasite (black arrow in C). L, lysosomes; P, parasite; F, flagellum. Scale bars: (A, C and D) 1 µm, (B) 200 nm.

**Figure 8:**
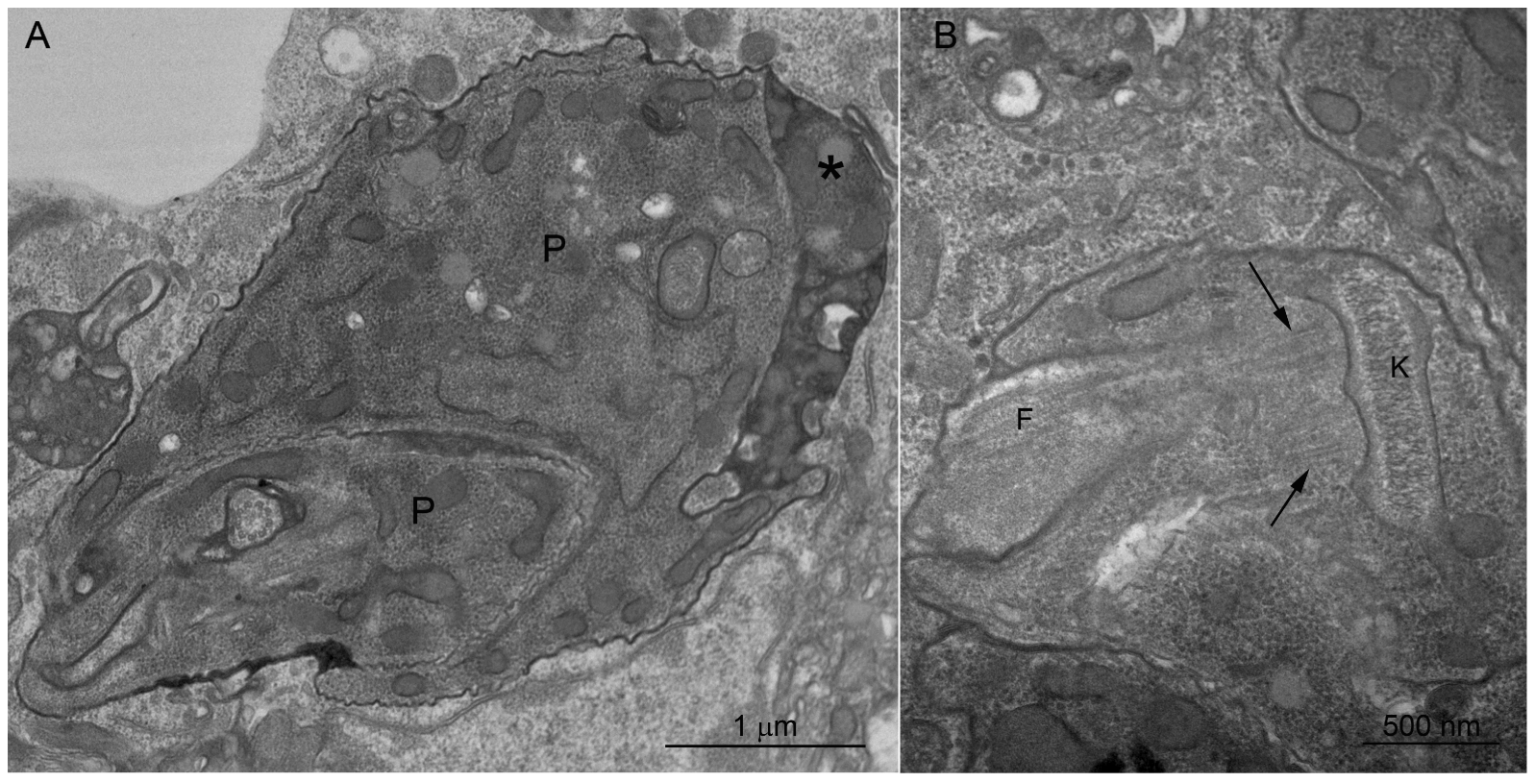
Multiple *P.* *serpens* parasites inside the PV. Infected hemocytes were analyzed at 72 hpi. (A) At least two parasites were observed within a vacuole (asterisk). (B) A parasite dividing as demonstrated by the presence of two basal corpuscles indicated by the arrows. P, parasite; F, flagellum; K, kinetoplast. No parasites being digested were observed at 72 hpi. Scale bars: (A) 1 µm, (B) 500 nm.

To identify the precise localization of internalized parasites inside hemocytes, we followed the internalization process using transmission electron microscopy (Fig. 7). After 2 hpi, parasites were observed inside a vacuole resembling parasitophorous vacuoles (PV) of other systems. These vacuoles were identified due to the presence of a continuous membrane around the parasite; we usually found only one parasite per vacuole (Fig. 7A). After 6 h, lysosome-like organelles, which are characterized by the presence of multiple vesicles and concentric membranes resembling myelin figures immersed in an electron-dense matrix, fused with the PV. (Fig. 7B and 7C). To confirm the lysosomal characteristics of some compartments, we pre-incubated hemocytes with gold-labeled BSA. Different cytoplasmic compartments were labeled (Fig. 7C and 7D). At 72 hpi, the parasites remained located within the PV. However, we observed a greater number of parasites per PV, thereby suggesting that they not only survive within the PV, but they could also be able to multiply there (Fig. 8A and 8B).

### *P. serpens* reaches the *O. fasciatus* salivary glands

**Figure 9:**
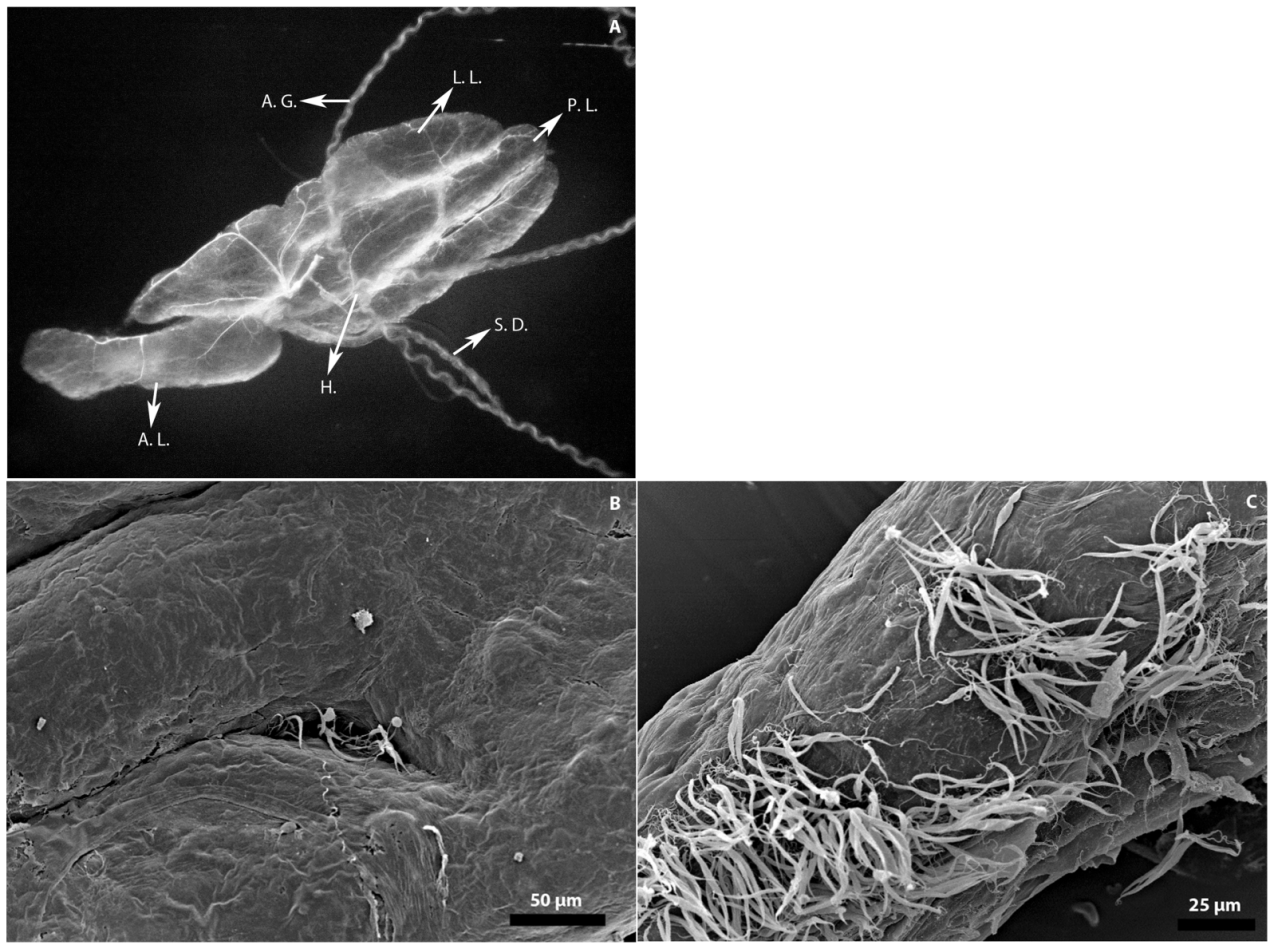
*P.* *serpens* reaches the salivary gland of *O. fasciatus*. (A). Anatomy of the *O. fasciatus* salivary gland. The arrows show: lateral lobes (L.L.), posterior lobes (P.L.), anterior lobes (A.L.), accessory glands (A.G.), salivary duct (S.D.) and hilus (H). (B). SEM of infected salivary glands at 24 hpi. (C) Salivary glands infected with long slender forms at 72 hpi.

An important step in the life cycle of *P. serpens* is the recognition of, binding to and invasion of *O. fasciatus* salivary glands (Fig. 9A). We investigated whether *P. serpens* reaches and recognizes the *O. fasciatus* salivary glands despite the cellular responses toward *P. serpens*. Using the SEM approach, parasites where observed adhering to the salivary glands at 24 hpi (Fig. 9B). The number of adhered parasites increased, with the highest density observed at 72 hpi (Fig. 9C), suggesting the immune response to *P. serpens* is not sufficient to interrupt the parasitic life cycle.

## Discussion

To complete its life cycle within the vector, the *Phytomonas* species must successfully survive the immune response of the vector. Once the parasite reaches the hemocoel, it must cope with one of the major players of the immune system, hemocytes. In a broad sense, cells respond to infection by forming nodules, phagocytizing microorganisms, producing antimicrobial peptides and producing the pro-enzyme pro-phenoloxidase. In the present study, using a model of systemic infection, we have described the interaction between *P. serpens* and *O. fasciatus* hemocytes. In agreement with previous observations of both the natural infection of the vector *Phthia picta* and the experimental infection of *O. fasciatus* with *P. serpens*, the parasite survives the invasion of the hemocoel and reaches the salivary gland [4], [25], a condition common to the life cycle of parasites that develop in the hemolymph within their respective vector, such as *Plasmodium* species and *T. rangeli* [26], [27]. This mode of infection is widely used for studying the interaction of trypanosomatids that invade the hemocoel of their insect hosts, especially in the model of interaction between *T. rangeli* with *R. prolixus* [28], [29].

Earlier studies had demonstrated the presence of only two types of hemocytes in *O. fasciatus*, plasmatocytes and oenocytes [30]. Later on, prohemocytes and granulocytes were also observed in these insects [31]. On the other hand, both authors agree that the vast majority of the *O. fasciatus* hemocytes are phagocytic cells [31]. The plasmatocytes are highly variable, as they have an expressive number of globular inclusion bodies of differing electron density and size [31]. In this report we show that hemocytes respond to the systemic infection caused by *P. serpens* with both types of responses: nodulation and phagocytosis. Phagocytosis takes place during the entire time course of the infection. Additionally, the number of parasites within hemocytes increased during the same period. The increase of the number of parasites inside hemocytes indicates a multiplication within these cells, as previously reported for *T. rangeli* [32]. This light microscopical observation is supported by TEM. Although lysosomes fused with the parasitophorous vacuole, the parasites survived and divided, a phenomenon also evident in the interaction between *R. prolixus* and *T. rangeli* [12], [33].

In mosquitoes infected with *Plasmodium*, only 10 to 20% of the sporozoites released from the midgut oocysts reach the salivary glands [34]. In our experimental model, we were not able to determine the extent of *P. serpens* killing. We did not observe any dead parasites in the hemolymph. In contrast, some parasites were observed in the process of cell division in the hemolymph. We used parasites in the stationary phase to perform the infection experiments; therefore, the ability to multiply in the hemolymph may be an intrinsic property of these organisms. *T. rangeli*, similar to *P. serpens*, multiplies freely in *R. prolixus* hemolymph [33].

In this report, despite the cellular responses towards the parasites, they manage to reach the salivary glands, as an increase in the number of parasites attached to the salivary glands is observed during the time course of infection. We have not observed parasites dividing at the salivary gland outer membrane, but we cannot disregard this possibility. The majority of the attached parasites were more than 60 µm in length; these parasites were referred to here as long slender forms. These long slender parasites have been documented previously in *Phytomonas*-infected insects [1]. Indeed, in a previous study describing the life cycle of *P. elmassiani* in *O. fasciatus*, an unequal fission of the long slender parasites has been reported to occur on the salivary glands [5]. Nonetheless, during the interaction observed between *P. serpens* and *P. picta* salivary glands using a TEM approach, parasites undergoing cellular division were not observed. The same long slender forms have been described before for *P. elmassiani* infecting *O. fasciatus* as well as for *P. serpens* during the colonization of both vectors *P. picta* and *Nezara viridula* [4], [35]. The function of such long slender forms in the parasite life cycle is completely understudied. *P. serpens* does not undergo differentiation during development, in other words, it remains as a promastigote form throughout its entire life cycle. This long slender form may help the parasite to avoid being phagocytized. Interesting, long slender *T. rangeli* epimastigotes have been described in the hemolymph of *R. prolixus*. Therefore, these common morphotypes might be important for hemolymph colonization. Further studies are required to determine the morphological changes *P. serpens* parasites undergo during their life cycle, in order to understand the function of these long slender flagellates. In conclusion, this work examines the *O. fasciatus* hemocyte response towards *P. serpens* during a systemic parasite infection. To the best of our knowledge, this is the first description of the interaction of *Phytomonas* with insect hemocytes. Taken together, our data suggest that *P. serpens* reproduces in *O. fasciatus* hemolymph, survives the hemocyte response and complete its life cycle by reaching the salivary glands.

## Materials and Methods

### Insects

A culture kit of *O. fasciatus* (milkweed bug) was purchased from Carolina Biological Supply Company, Burlington, North Carolina, USA. These insects founded the colony we maintain in our laboratory in plastic pitchers under a 12 h light/dark cycle at 28°C with 70–80% relative humidity [25].

### Parasites

*P. serpens* parasites (isolate 9T, CT-IOC-189) isolated from tomato (*Lycopersicon esculentum*) were provided by Dr. Maria A. de Sousa, the Trypanosomatid Collection, Instituto Oswaldo Cruz, Rio de Janeiro, Brazil. The parasites were maintained by weekly transfer in Warren medium (37 g/l brain-heart infusion, 1 mg/l folic acid and 10 mg/l hemin) supplemented with 10% fetal calf serum at 26°C. The parasites were harvested at stationary growth phase via centrifugation for 10 min at 1,500× *g* at 4°C and washed three times with PBS (phosphate buffered saline) containing 137 mM NaCl, 2.7 mM KCl, 1.4 mM KH_2_PO_4_ and 10 mM Na_2_HPO_4_, pH 7.2. Cellular viability was assessed by motility prior to insect injections. The viability of the parasites was never affected by the conditions used in this study.

### Insect experimental infection and parasite quantification

For experimental infection, the insects were immobilized on ice and 4 µl of PBS containing 5×10^4^ parasites was inoculated with a Hamilton pipette laterally between the first and second thoracic segments. Alternatively, the insects were mock challenged with PBS. The challenged insects were maintained as described above. The density of parasites in the hemolymph was determined by counting the parasites in an improved Neubauer hemocytometer chamber. A total of 60 insects were used in this experiment both for experimental infection and mock challenge.

### Extraction of hemolymph and quantification of hemocytes

Non-challenged, mock-challenged and challenged insects were used for this experiment. The hemolymph was collected at 6, 24, 48 and 72 hpi by clipping off the first two pairs of insect legs and the pair of antenna. The hemolymph was kept in 1.5 ml plastic tubes at 4°C. The hemocytes were quantified in an improved Neubauer hemocytometer chamber. The hemocyte count was compared using the nonparametric Student's *T*-test with GraphPad Prism version 5.00 for Windows, GraphPad Software, San Diego, California, USA.

### In vivo gold-labeled BSA uptake assay

Insects were immobilized on ice and gold-labeled BSA particles solution (10 nm diameter) diluted 1∶5 in PBS was injected using the same method applied for parasite injection (described above). After 30 min, the insects were challenged with *P. serpens* as previously described. After 2, 6 and 72 hpi the hemolymph was extracted; the hemocytes were then collected via centrifugation at 1,500× *g* and washed twice in PBS. Then, the cells were processed for transmission electron microscopy as described below.

### Dissection of salivary glands

The salivary glands were dissected by carefully pulling the head and the first thoracic segment out of the insect body. The glands were rinsed three times in cold PBS at 4°C and immediately used for the experiments.

### Differential interferential contrast (DIC) microscopy and Giemsa staining of hemocytes infected with *P. serpens*

For fresh observation of hemocytes, hemolymph was collected as described above and drops were spread onto microscopy slides, subsequently covered with a coverslip and immediately examined via DIC microcopy [36]. Alternatively, the hemolymph was left to dry on the microscopy slides, ethanol fixed and then stained with Giemsa for 15 min. Coverslips were added onto the glass slides with Permount (Fisher Scientific, NJ, USA). Alternatively, the samples were air-dried, methanol fixed and stained with Giemsa. The slides were photographed using a Zeiss Axioplan 2 light microscope (Oberkochen, Germany) equipped with a Color View XS digital video camera. The percentage of infected hemocytes was determined by counting 200 Giemsa-stained hemocytes.

### Ultrastructural analysis of salivary glands using scanning electron microscopy (SEM)

Salivary glands collected at 6, 24, 48 and 72 hpi were washed twice with TBS (20 mM Tris and 150 mM NaCl, pH 7.6) and fixed in 2.5% glutaraldehyde, 5 mM calcium chloride and 2% sucrose in 0.1 M cacodylate buffer (pH 7.2) for 1 h at room temperature. The salivary glands were then washed with TBS, dehydrated in graded ethanol and then critical point dried with CO_2_. The samples were allowed to adhere to scanning electron microscopy stubs and then coated with a 20-nm thick gold layer in a sputtering device. The salivary glands were examined using a JEOL JSM 5310 scanning 185 electron microscope (Tokyo, Japan) operating at 25 kV.

### Ultrastructural analysis of *P. serpens*-infected hemocytes using transmission electron microscopy (TEM)

The hemolymph of non-infected and infected insects was collected as described above and fixed in 2.5% glutaraldehyde, 5 mM calcium chloride, and 2% sucrose in 0.1 M cacodylate buffer (pH 7.2) for 1 h at room temperature. The samples were rinsed in 0.1 M cacodylate buffer (pH 7.2) containing 2% sucrose and post-fixed in 1% osmium tetroxide (O_S_O_4_), 0.8% potassium ferrocyanide and 5 mM calcium chloride in 0.1 M cacodylate buffer (pH 7.2) for 1 h at room temperature. They were then dehydrated in graded acetone and embedded in PolyBed 812 epoxy resin (Polysciences Inc., Warrington, PA, USA). Ultrathin sections obtained using a Leica ultramicrotome (Nussloch, Germany) were stained with uranyl acetate and lead citrate and subsequently observed using a FEI Morgagni F268 transmission electron microscope (Eindhoven, The Netherlands) operating at 80 kV.

### Statistics

The statistical significance between the samples and controls is indicated in the figure legends. Survival curve comparisons were made via log-rank analysis using GraphPad Prism version 5.00 for Windows. Statistical analysis of parasite length size, hemocyte abundance and hemocyte containing phagocytized parasites was performed using One-way ANOVA with Dunnett's post-hoc test using GraphPad Prism version 5.00 for Windows, GraphPad Software, San Diego, California, USA.
